# Assessment of strategies for switching patients from olanzapine to risperidone: A randomized, open-label, rater-blinded study

**DOI:** 10.1186/1741-7015-6-17

**Published:** 2008-06-30

**Authors:** Rohan Ganguli, Jaspreet S Brar, Ramy Mahmoud, Sally A Berry, Gahan J Pandina

**Affiliations:** 1 Western Psychiatric Institute and Clinic, O'Hara Street, Pittsburgh, PA 15213-2593, USA; 2 Ethicon, Inc., US Highway 22 West, Somerville, NJ 08876, USA; 3 Johnson & Johnson Pharmaceutical Research & Development, LLC, Trenton-Harbourton Road, Titusville, NJ 08560, USA; 4 Ortho McNeil Janssen Scientific Affairs, LLC, Trenton-Harbourton Road, Titusville, NJ 08560, USA; 5 University of Toronto, Center for Addiction and Mental Health, Toronto, ON M6J1H4, Canada

## Abstract

**Background:**

In clinical practice, physicians often need to change the antipsychotic medications they give to patients because of an inadequate response or the presence of unacceptable or unsafe side effects. However, there is a lack of consensus in the field as to the optimal switching strategy for antipsychotics, especially with regards to the speed at which the dose of the previous antipsychotic should be reduced. This paper assesses the short-term results of strategies for the discontinuation of olanzapine when initiating risperidone.

**Methods:**

In a 6-week, randomized, open-label, rater-blinded study, patients with schizophrenia or schizoaffective disorder, on a stable drug dose for more than 30 days at entry, who were intolerant of or exhibiting a suboptimal symptom response to more than 30 days of olanzapine treatment, were randomly assigned to the following switch strategies (common risperidone initiation scheme; varying olanzapine discontinuation): (i) abrupt strategy, where olanzapine was discontinued at risperidone initiation; (ii) gradual 1 strategy, where olanzapine was given at 50% entry dose for 1 week after risperidone initiation and then discontinued; or (iii) gradual 2 strategy, where olanzapine was given at 100% entry dose for 1 week, then at 50% in the second week, and then discontinued.

**Results:**

The study enrolled 123 patients on stable doses of olanzapine. Their mean age was 40.3 years and mean (± standard deviation (SD)) baseline Positive and Negative Syndrome Scale (PANSS) total score of 75.6 ± 11.5. All-cause treatment discontinuation was lowest (12%) in the group with the slowest olanzapine dose reduction (gradual 2) and occurred at half the discontinuation rate in the other two groups (25% in abrupt and 28% in gradual 1). The relative risk of early discontinuation was 0.77 (confidence interval 0.61–0.99) for the slowest dose reduction compared with the other two strategies. After the medication was changed, improvements at endpoint were seen in PANSS total score (-7.3; *p *< 0.0001) and in PANSS positive (-3.0; *p *< 0.0001), negative (-0.9; *p *= 0.171) and anxiety/depression (-1.4; *p *= 0.0005) subscale scores. Severity of movement disorders and weight changes were minimal.

**Conclusion:**

When switching patients from olanzapine to risperidone, a gradual reduction in the dose of olanzapine over 2 weeks was associated with higher rates of retention compared with abrupt or less gradual discontinuation. Switching via any strategy was associated with significant improvements in positive and anxiety symptoms and was generally well tolerated.

**Trial registration:**

ClinicalTrials.gov NCT00378183

## Background

In clinical practice the successful treatment of schizophrenia may require switching between antipsychotics [1]. The reasons for changing treatments include inadequate or complete lack of efficacy, partial compliance or noncompliance with medication, and the presence of adverse events such as movement disorders, weight gain, somnolence, endocrine side effects, and metabolic dysfunction. The possibility of exacerbation of psychotic symptoms due to withdrawal of the original antipsychotic before the new antipsychotic has become effective is a major consideration [1-4]. Conversely, when drugs with long half-lives are quickly replaced with new treatments, consideration must be given to possible excessive drug effects.

Guidelines on changing a patient's antipsychotic medications were provided by a Consensus Study Group in 1996 [5]. Their primary purpose was to assist clinicians in switching patients from conventional antipsychotics and from clozapine to the new antipsychotic risperidone. The guidelines suggest that for hospitalized schizophrenia patients who are at low risk for psychotic exacerbation or aggressive or suicidal reactions and who are receiving antipsychotics other than clozapine, the previous antipsychotic can be withdrawn completely before risperidone is started. Gradual withdrawal while introducing the other antipsychotic, however, may be necessary in patients who are combative or assaultive, those with a history of exacerbation of psychotic behavior or suicidal tendencies when the dose of an antipsychotic was reduced, those receiving high doses of an antipsychotic, and those receiving clozapine (withdrawal of clozapine has been associated with withdrawal symptoms and rebound psychosis in some patients).

A number of studies have assessed methods for changing treatments in patients with schizophrenia, with varying results. In 1998, Henderson et al [6] reported the results of a study of 19 outpatients with treatment-refractory schizophrenia who were switched from clozapine to olanzapine. Olanzapine was started at 5 mg/day and slowly increased to a maximum of 30 mg/day. After 1 week, clozapine was gradually withdrawn in increments of 25 to 50 mg per week. Eight of the 19 patients were rated as responders. Seven patients decompensated seriously and had to be hospitalized and restabilized on clozapine. In four additional patients, symptoms worsened and the olanzapine was slowly replaced with clozapine.

In a study of clozapine responders by Littrell et al [7], 20 outpatients were gradually switched from clozapine to olanzapine. In most cases this was necessitated because of the adverse events experienced with clozapine. Olanzapine was introduced at 5 mg/day and over a 2-week period the clozapine dose was reduced in increments of 25 mg every other day and the olanzapine dose increased. Two of the patients asked that clozapine be reinstituted because of symptom exacerbation. In the other patients, symptom improvement was maintained and the severity of adverse events was reduced.

Takahashi et al [8,9] have studied treatment changes in two groups of first-episode schizophrenia patients (inpatients and outpatients) who had failed to respond to treatment. One group was switched from olanzapine to risperidone (*N *= 51) and the other from risperidone to olanzapine (*N *= 58). In both studies, the treatment change was made over a 2-week period during which the two antipsychotics were gradually replaced. A treatment response in these 12-week studies was defined as a 20% reduction in Brief Psychiatric Rating Scale total scores and a final Clinical Global Impressions (CGI) score of 3 (mildly ill). In the olanzapine-risperidone study, 35% of the patients were rated as responders and in the risperidone-olanzapine study 29% were rated as responders. Treatment was generally well tolerated. All of the above studies report a single method employed to change existing antipsychotic treatment to another antipsychotic. They do not provide a comparison of methods to change antipsychotic medication in patients with psychotic illnesses.

The purpose of the present study of patients with schizophrenia or schizoaffective disorder was to evaluate the efficacy and safety of three strategies for switching antipsychotic treatment. This 6-week study was phase 1 of a longer-term study that addressed weight-control strategies and the prevalence of the metabolic syndrome in the patients switched from olanzapine to risperidone [10,11]. The results of phase 2 of the study are also summarized below.

## Methods

The 6-week, open-label, rater-blinded study was designed to evaluate the efficacy and safety of three strategies for switching patients with schizophrenia or schizoaffective disorder from olanzapine to risperidone. The reason for switching treatments in these patients was the lack of an adequate clinical response to olanzapine, a body mass index (BMI) of more than 26 kg/m^2 ^or glucose dysregulation.

Patients were randomly assigned to one of three strategies for switching from olanzapine to risperidone, abrupt, gradual 1 (1 week), or gradual 2 (2 weeks), as described below. Dose adjustments were made in an open-label fashion; however, all psychiatric assessments were performed by raters blinded to study group assignment. The trial was conducted in accordance with current International Conference on Harmonisation of Technical Requirements for Registration of Pharmaceuticals for Human Use (ICH) Good Clinical Practice guidelines and the Declaration of Helsinki (World Medical Association).

### Patients

Patients were recruited at 19 sites in the United States. Patients were men or women aged 18 to 65 years with a DSM-IV (Diagnostic and Statistical Manual of Mental Disorders) diagnosis of schizophrenia or schizoaffective disorder. Outpatients or stable chronic inpatients could be included. All patients were to have received a stable dose of olanzapine for at least 30 days before randomization and to have had no acute exacerbation of psychotic symptoms within the preceding 3 months. To qualify for the study subjects also had to meet the following criteria: a Positive and Negative Syndrome Scale (PANSS) [12] total score of 60 to 120, a BMI above 26 kg/m^2^, type 2 diabetes, or laboratory abnormalities related to glucose metabolism, including fasting plasma glucose above 80 mg/dl or an oral glucose tolerance test 2-hour value above 139 mg/dl.

Exclusion criteria included previous treatment failure with risperidone, significant adverse event or sensitivity related to risperidone, treatment-refractory schizophrenia or schizoaffective disorder, antipsychotic treatment other than olanzapine in the 30 days preceding randomization, mental retardation, substance dependence (DSM-IV), or serious or unstable concomitant nonpsychiatric medical illness.

Written informed consent was obtained from each patient or a relative, guardian, or legal representative.

### Medication

**Figure 1:**
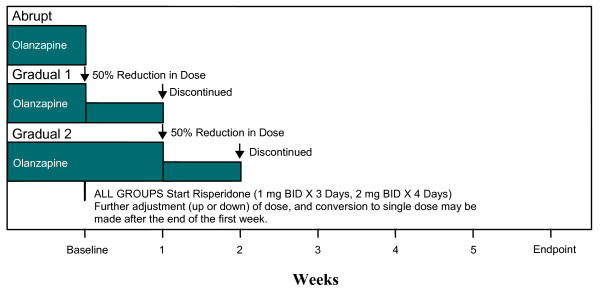
Dosing schedules for the three strategies.

The oral dose of risperidone for all patients in all three strategies was 1 mg twice daily for the first 3 days, followed by 2 mg twice daily for the next 4 days. Further dose adjustments or conversion to a single daily dose could be made at the end of the first week according to the investigator's judgment. Patients were randomly assigned to discontinue olanzapine according to one of the following three treatment strategies (Figure 1):

(1) Abrupt: olanzapine was discontinued on the day of risperidone initiation (beginning of week 0).

(2) Gradual 1: the study entry dose of olanzapine was reduced by half (or as close to half of the starting dose as possible) on the day of risperidone initiation. The reduced dose of olanzapine was given for 1 week and then discontinued. The risperidone dose was adjusted as described above.

(3) Gradual 2: the study entry dose of olanzapine remained unchanged for the first week of the study while risperidone was initiated and adjusted as described above. At the end of the first week, the dose of olanzapine was reduced to half of the study entry dose (or as close to half of the starting dose as possible) and then discontinued at the end of the second week.

Any other medication prescribed at a steady dose for at least 3 weeks before randomization could be continued during the trial. Any psychotropic medication other than antipsychotics prescribed before randomization was continued at the study entry dose throughout the trial. The only psychotropic medications that could be introduced during the study period were lorazepam (at most 12 mg/day) for agitation or anxiety and zolpidem tartrate (at most 10 mg/day) for insomnia. Medications prescribed before the trial to control movement disorders could be discontinued or the dose reduced or increased. Benztropine or propranolol could be initiated to control reversible movement disorders if clinically necessary.

### Assessments

The primary efficacy assessment was the 30-item PANSS, completed at baseline and weeks 1 through 6. Total PANSS scores are reported, as well as scores on three PANSS subscales (positive and negative symptoms and anxiety/depression). The latter includes the items anxiety, guilty feelings, tension, and depression (items 2, 3, 4, and 6) from the PANSS general psychopathology subscale. Additional efficacy assessments were the CGI Change (CGI-C) and Severity (CGI-S) scales [13]; the Drug Attitude Inventory [14] (DAI-10) completed at baseline and weeks 1 through 6; and the Global Assessment of Functioning (GAF) [15] at baseline and weeks 1 through 6. The DAI-10 is a 10-item scale used to assess the patient's view of the use of psychiatric medications and his or her experience when taking these drugs.

Weight was measured at baseline and weekly throughout the study period. Other anthropometric measurements (height, BMI, slenderness index (height in meters divided by the sum of the waist and hip width in meters), waist circumference, waist-to-hip ratio) were made or calculated at baseline and week 6.

Adverse events and vital signs were recorded at each visit. Laboratory tests, including hematology, biochemistry, lipid profile, plasma insulin, glycosylated hemoglobin, serum leptin, and abbreviated glucose tolerance test, were performed at baseline and week 6.

### Statistical analysis

Safety and efficacy analyses were completed on all randomized patients who received at least one dose of study medication. All statistical tests were interpreted at the 5% significance level (two-tailed). Within-group differences were assessed using a paired *t*-test. Between-group differences were assessed using chi-square or Fisher's exact tests as appropriate for categorical variables and two-way analysis of variance (ANOVA) with the treatment group and site as factors for continuous variables. For continuous change scores, between-group comparisons were calculated using analysis of covariance (ANCOVA) models, with the treatment group and site as the fixed effect design factors and baseline scores as the covariate. No adjustment for multiple comparisons was made.

## Results

A total of 123 patients, all outpatients, were enrolled (41 in the Abrupt group, 40 in the Gradual 1 group, and 42 in the Gradual 2 group). Two patients did not receive any study medication, 1 in the Abrupt group and 1 in the Gradual 1 group. The safety data set included all patients who received at least one dose of study medication. The intention-to-treat efficacy set included all patients who received at least one dose of study medication and at least one completed post-baseline evaluation.

Patient disposition is shown in Table 1 (safety population). Significantly more patients in the abrupt (25%) and gradual 1 (28%) groups discontinued the study prematurely than in the gradual 2 group (12%); relative risk = 0.833, confidence interval (CI) = 0.70–0.99, *p *= 0.039.

**Table 1:** Patient disposition by treatment group.

	**Abrupt**	**Gradual 1**	**Gradual 2**	**All treatments**
Randomized, *n*	41	40	42	123
Safety population, *n*	40	39	42	121
Completed, %	75	72	88	79
Discontinued, %	25	28	12	21
Adverse event	5	15	5	8
Withdrew consent	10	8	0	6
Insufficient response	5	0	0	2
Lost to follow-up	0	3	2	2
Noncompliance	0	0	2	1
Other	5	3	2	2

The safety population includes all randomized patients who received at least one dose of study medication. Between-group discontinuation differences were not significant (*p *= 0.1635).

Patient characteristics at baseline were generally similar in the three groups, with the Abrupt group having slightly higher BMIs and the Gradual 2 group more likely to have a diagnosis of schizoaffective disorder (Table 2).

**Table 2:** Baseline patient demographic and clinical characteristics

	**Abrupt (*n *= 37)**	**Gradual 1 (*n *= 39)**	**Gradual 2 (*n *= 40)**
Sex, % female	51	51	50
Race/ethnicity, %			
White	57	54	53
Black	32	31	33
Hispanic	5	13	8
Asian	5	3	5
Age, mean (± SD) years	41.6 (± 10.2)	41.5 (± 10.4)	40.3 (± 9.1)
BMI, mean (± SD) kg/m^2^	36.2 (± 9.1)	34.0 (± 6.2)	32.3 (± 4.7)
Diagnosis, %			
Schizophrenia	57	56	53
Schizoaffective disorder	43	44	47
Olanzapine dose, mean (± SD) mg/day	14.4 (± 5.4)	15.5 (± 6.3)	16.4 (± 7.9)

### Exposure to medication

Mean (± SD) doses of olanzapine at study entry were 14.4 (± 5.4), 15.5 (± 6.3), and 16.4 (± 7.9) mg/day in the abrupt, gradual 1, and gradual 2 groups, respectively. The mean (± SD) modal doses of risperidone at endpoint were 4.3 (± 1.3), 4.9 (± 1.7), and 4.4 (± 1.6) mg/day in the abrupt, gradual 1, and gradual 2 groups, respectively. Mean (± SD) modal doses of risperidone over the entire 6-week study period were 4.2 (± 1.4), 4.6 (± 1.4), and 4.1 (± 1.4) mg/day in the three groups.

The most common concomitant medications initiated during the study were sedative/hypnotic drugs in 12 (30%), 12 (30%), and 9 (21%) patients in the abrupt, gradual 1, and gradual 2 groups, respectively (*p *= 0.0145, abrupt versus gradual 2; chi-square test). Antiparkinsonism drugs were received by two (5%), two (5%), and three (7%) patients in the three groups.

### Efficacy

**Figure 2:**
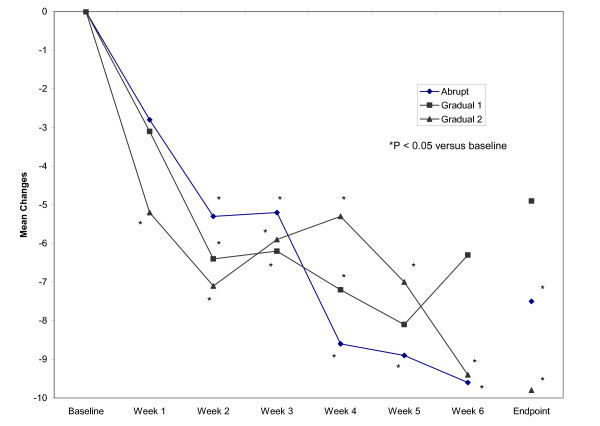
Improvements in PANSS total scores from baseline to endpoint in the three treatment groups.

**Figure 3:**
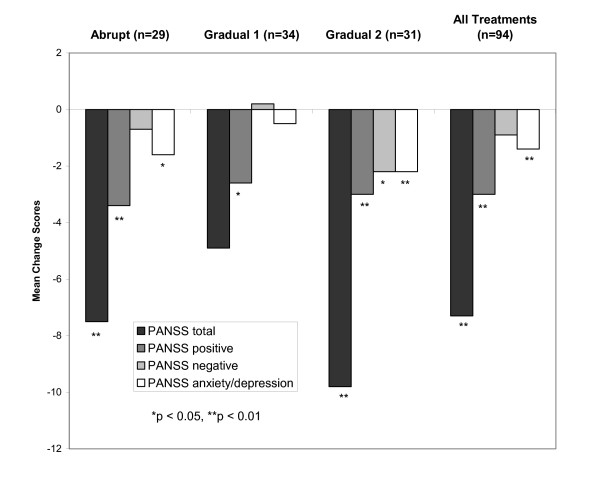
Mean changes from baseline in PANSS total and subscale scores.

In this 6-week study, improvements in PANSS scores were seen in each of the three groups of patients (Table 3, Figure 2). In the abrupt group, significant improvements from baseline to endpoint were seen in PANSS total scores and scores on the positive and anxiety/depression subscales (Figure 3). In the gradual 1 group, a significant improvement from baseline to endpoint was seen only in PANSS positive scores. In the gradual 2 group, significant improvements from baseline to endpoint were seen in all evaluated PANSS domains. Moreover, at week 1 a significant improvement in the PANSS total score was noted only in the gradual 2 group (*p *= 0.0022); see Figure 2. Between-group comparisons were not statistically significant.

**Table 3:** Mean (± SD) baseline and changes at endpoint in PANSS total, positive, negative, and anxiety/depression scores in the three groups of patients.

**PANSS**	**Abrupt (*n *= 29)**	**Gradual 1 (*n *= 34)**	**Gradual 2 (*n *= 31)**	**All Groups (*n *= 94)**	**Between-treatment *p*-value**^**a**^
Total					
Baseline	74.6 (± 10.6)	76.0 (± 12.3)	76.2 (± 11.6)	75.6 (± 11.5)	
Change	-7.5 (± 14.4)†	-4.9 (± 17.1)	-9.8 (± 15.1)‡	-7.3 (± 15.6)‡	0.1497
Positive					
Baseline	21.7 (± 4.2)	23.1 (± 5.2)	21.1 (± 5.5)	22.0 (± 5.1)	
Change	-3.4 (± 4.6)‡	-2.6 (± 6.4)*	-3.0 (± 4.9)†	-3.0 (± 5.4)‡	0.2009
Negative					
Baseline	17.9 (± 4.8)	18.1 (± 5.2)	19.5 (± 5.7)	18.5 (± 5.2)	
Change	-0.7 (± 6.0)	0.2 (± 6.2)	-2.2 (± 5.8)*	-0.9 (± 6.1)	0.2904
Anxiety/depression					
Baseline	11.1 (± 3.4)	10.3 (± 3.6)	10.9 (± 3.4)	10.7 (± 3.5)	
Change	-1.6 (± 3.6)*	-0.5 (± 3.6)	-2.2 (± 3.9)†	-1.4 (± 3.7)‡	0.618

^a^From an analysis of covariance (ANCOVA) model with treatment, site, and baseline value as factors.

*p < 0.05, †*p *< 0.01, ‡*p *< 0.001 versus baseline (paired *t*-test).

The proportions of patients who were improved on the CGI-C at endpoint were 50%, 44%, and 54% in the abrupt, gradual 1, and gradual 2 groups, respectively. Patients who were worse at endpoint included 22%, 26%, and 13% of the abrupt, gradual 1, and gradual 2 groups, respectively. The proportions of patients with no symptoms or slight or mild symptoms on the CGI-S scale were 38% of the abrupt group, 47% of the gradual 1 group, and 40% of the gradual 2 group at baseline, and 64%, 44%, and 67%, respectively, at endpoint. Between-group differences in CGI scores were not significant.

The GAF scores improved significantly from baseline to endpoint in the abrupt, gradual 2, and pooled treatment groups, but not in the gradual 1 group. Mean (± SD) changes in GAF scores at endpoint were 4.3 (± 7.5; *p *= 0.0016), 0.0 (± 9.6; *p *= NS), and 3.6 (± 10.8; *p *= 0.0449) in the abrupt, gradual 1, and gradual 2 treatment groups, respectively. Between-group comparisons were not significant at endpoint.

The mean DAI-10 scores were reduced in the abrupt group from baseline to endpoint and improved significantly in the gradual 2 group, in whom the mean (± SD) change at endpoint was 1.3 (± 3.2; *p *= 0.0120). Between-group differences were not significant.

### Safety

Adverse events were generally reported to be mild. Adverse events were reported in 73%, 90%, and 79% of the abrupt, gradual 1, and gradual 2 groups, respectively; these were mild or moderate in 93%, 89%, and 91% of patients who experienced an adverse event. Adverse events reported in at least 10% of patients in any treatment group were insomnia, anxiety, sedation, somnolence, headache, and aggravated psychosis (Table 4). The overall between-group difference in the incidence of aggravated psychosis was significant (*p *= 0.0483, exact test). The incidence of movement disorders was low in the three treatment groups throughout the 6-week study. Discontinuation because of an adverse event was reported in 5%, 15%, and 5% in the abrupt, gradual 1, and gradual 2 groups, respectively (Table 1). Discontinuations because of adverse events during the first 2-week transition period were low in all three study groups, with one each in the abrupt and gradual 2 groups and two in the gradual 1 group. The incidence of movement disorders was low in all three groups during the first 2 weeks, with one report in each group.

**Table 4:** Adverse events reported in at least 10% of patients in any treatment group.

	**Abrupt (*n *= 40)**	**Gradual 1 (*n *= 39)**	**Gradual 2 (*n *= 42)**	**All treatments (*n *= 121)**
Patients with any adverse event	73%	90 %	79%	80%
Insomnia	23%	13%	24%	20%
Anxiety	18%	13%	14%	15%
Sedation	5%	18%	12%	12%
Somnolence	10%	10%	10%	10%
Headache	3%	10%	7%	7%
Aggravated psychosis	13%	0	5	6%

No significant changes from baseline to week 6 or endpoint were seen in vital signs, body weight, or anthropometric measurements in any treatment group, with the exception of a significant reduction in mean standing diastolic blood pressure at week 6 and endpoint in the abrupt group only (-6.6 mmHg at week 6, *p *= 0.005; and -5.5 mmHg at endpoint, *p *= 0.0167; paired *t*-test). In the gradual 1 and 2 groups the respective values were -1.6 and 0.1 mmHg and 2.2 and 2.2 mmHg.

Significant improvements from baseline to endpoint in levels of total cholesterol (-8.22 mg/dl; *p *= 0.0075), very-low-density lipoprotein (-4.11 mg/dl; *p *= 0.0359), apolipoprotein A1 (-4.32 mg/dl; *p *= 0.0372), apolipoprotein B (-8.42 mg/dl; *p *< 0.0001), and triglycerides (-20.5 mg/dl; *p *= 0.0364) were seen in the pooled patient group (paired *t*-test). These data have been discussed in more detail elsewhere [11].

### Behavioral therapy for weight loss and the metabolic syndrome (phase 2)

After participating in the 6-week study of switching strategies (phase 1), all patients with a BMI above 26 kg/m^2 ^were invited to enroll in a weight-loss program while receiving risperidone [10]. Seventy-one consenting subjects were randomly assigned to 14 weeks of a behavioral treatment program for weight reduction (*n *= 34) or usual clinic care (*n *= 37). After 14 weeks, significant weight loss was reported in both treatment groups: mean (± SD) changes were -2.0 ± 3.8 kg in the subjects enrolled in the behavioral program and -1.1 ± 3.1 kg in the control group (both *p *< 0.05). The significant reductions in PANSS scores noted in the 6-week phase 1 were maintained during the 14 weeks [10]. The mean modal doses of risperidone were 4.3 mg/day at the end of phase 1 and 4.5 mg/day during phase 2.

In the 71 patients for whom data were available for both phases, the incidence of the metabolic syndrome was significantly reduced over the 20-week period [11]. The syndrome was identified in 38 (54%) of the patients at baseline and in 26 (37%) at the end of phase 2 (*p *< 0.0001). Significant reductions (*p *< 0.05) were also seen in mean (± SD) weight (-1.3 ± 4.9 kg), BMI (-0.7 ± 1.7 kg/m^2^), waist circumference (-1.6 ± 5.6 cm), and systolic (-4.7 ± 14.0 mmHg) and diastolic (-3.5 ± 11.0 mmHg) blood pressure [11].

## Discussion

In overweight/obese patients who had shown a poor response to treatment with olanzapine, the strategy involving the slowest dose reduction of previous medication (gradual 1) was associated with the highest rates of retention ('successful switching') compared with the other two strategies. Twelve percent of the gradual 1 group discontinued treatment prematurely, compared with 25% of the patients in the abrupt group and 28% in the gradual 2 group. The three methods of switching to risperidone were generally associated with improvements in symptoms and were relatively well tolerated.

Although improvements on the PANSS scale were noted in all three treatment groups, some differences in patterns of response were noted. Patients in the gradual 2 group showed significant improvements in PANSS total scores as early as week 1 (Figure 2) and significant improvements in scores on each of the three PANSS subscales (Figure 3). Patients in the abrupt group showed significant improvements in PANSS total scores and scores on two subscales, while patients in the gradual 1 group showed significant improvement on only one subscale. Between-group differences, however, were not significant.

Adverse events were generally mild. Some events that resemble withdrawal symptoms (nausea, vomiting, agitation, movement disorders) were reported. Except for anxiety, however, the incidence of such symptoms was low, and the time-course of these events did not clearly suggest olanzapine withdrawal as a cause. Nonetheless, some of the differences between treatment groups might be attributed to the different withdrawal schedules. The incidence of aggravated psychosis was highest in the abrupt group (13% versus 0% and 5% in the gradual 1 and gradual 2 groups, respectively), as would be consistent with abrupt withdrawal of an antipsychotic before optimum levels of the other antipsychotic are reached. A similar pattern was seen in the incidence of anxiety, although the differences were less marked (18% in the abrupt group versus 13% and 14% in the other two groups). In addition, the reduction in mean standing diastolic blood pressure reported only in the abrupt group may have been due to cholinergic rebound associated with abrupt withdrawal of olanzapine.

The incidence of study discontinuation due to adverse events was highest in the gradual 1 group (15% versus 5% in each of the other groups). It is unclear why a lower rate of discontinuation was not observed in the gradual 1 group, with more gradual discontinuation of olanzapine, than in the abrupt group, particularly as this strategy of brief overlap in treatments may be the most commonly used in clinical practice. The incidence of discontinuation due to withdrawal of consent was 0 in the gradual 2 group and 8% and 10% in the abrupt and gradual 1 groups, respectively.

Anxiety/depression and positive and total symptoms were improved when switching these patients from olanzapine to risperidone. The overall findings suggest that all three tested strategies of switching to risperidone would be reasonable approaches in patient care. However, the small group sample sizes make it hard to draw conclusions about individual strategies, especially when a consistent trend is not observed with increasingly gradual discontinuation of prior treatment. Thus, the apparent better efficacy and retention observed in the gradual 2 group is of uncertain significance. Further studies may be necessary to determine whether these apparent subtle differences between switch strategies persist over time and with a larger sample.

Our study was limited by its open-label design, although possible bias was mitigated by the fact that raters were blind to the switch strategy. Phase 1 of the study was also limited by its short duration, such that long-term effects of switching were not assessed. Further, switch strategies employed in the clinical practice setting often vary the duration of switch based upon the needs of the individual patient, and the current study was limited due to study-blinding procedures.

## Conclusion

Our study confirms that stable outpatients with schizophrenia or schizoaffective disorder who require an alternative treatment can be safely switched from olanzapine to risperidone and experience improvements in symptom control. Our results also suggest that rapid initiation of the new medication and very gradual withdrawal of the old medication may be more successful than more rapid withdrawal strategies.

## Competing interests

RG declares he has received research grants and consulting fees or honoraria from Astra Zeneca, Bristol Myers Squibb, Janssen, Lilly, Pfizer, and Solvay. JSB declares he has no relevant conflicts of interest. RM declares he is an employee of Ethicon, Inc. RM is a Johnson & Johnson (J&J) stockholder. SAB declares she is an employee of Johnson & Johnson Pharmaceutical Research & Development. SAB is a J&J stockholder. GJP declares he is an employee of Janssen, the company that funded the research, manuscript development, and the journal's article processing charge. GJP is a J&J stockholder.

## Authors' contributions

RG and JSB were involved in the study conception and design, analysis plan, data interpretation and manuscript revision. RM, SAB and GJP were involved in the study oversight and conduct, data analysis and interpretation, and manuscript development and revision. All authors read and approved the final manuscript.

## Pre-publication history

The pre-publication history for this paper can be accessed here:


